# A Proteomic Study of Atherosclerotic Plaques in Men with Coronary Atherosclerosis

**DOI:** 10.3390/diagnostics9040177

**Published:** 2019-11-07

**Authors:** Ekaterina M. Stakhneva, Irina A. Meshcheryakova, Evgeny A. Demidov, Konstantin V. Starostin, Evgeny V. Sadovski, Sergey E. Peltek, Michael I. Voevoda, Alexander M. Chernyavskii, Alexander M. Volkov, Yuliya I. Ragino

**Affiliations:** 1 Research Institute of Internal and Preventive Medicine - Branch of the Institute of Cytology and Genetics, Siberian Branch of Russian Academy of Sciences, 630089 Novosibirsk, Russia; stinger000@mail.ru (E.V.S.); mvoevoda@ya.ru (M.I.V.); ragino@mail.ru (Y.I.R.); 2 Institute of Cytology and Genetics, Siberian Branch of Russian Academy of Sciences, 630090 Novosibirsk, Russia; miren@ngs.ru (I.A.M.); scratch_nsu@ngs.ru (E.A.D.); kosten81@ngs.ru (K.V.S.); peltek@bionet.nsc.ru (S.E.P.); 3 The Federal State Budgetary Institution “National Medical Research Center named academician E.N. Meshalkin” of the Ministry of Health of the Russian Federation, 630055 Novosibirsk, Russia; amchern@mail.ru (A.M.C.); mail@meshalkin.ru (A.M.V.)

**Keywords:** atherosclerosis, proteomics, mass spectrometry, atherosclerotic plaque, tropomyosin

## Abstract

Background: To study the changes in protein composition of atherosclerotic plaques at different stages of their development in coronary atherosclerosis using proteomics. Methods: The object of research consisted of homogenates of atherosclerotic plaques from coronary arteries at different stages of development, obtained from 15 patients. Plaque proteins were separated by two-dimensional electrophoresis. The resultant protein spots were identified by the matrix-assisted laser desorption ionization method with peptide mass mapping. Results: Groups of differentially expressed proteins, in which the amounts of proteins differed more than twofold (*p* < 0.05), were identified in pools of homogenates of atherosclerotic plaques at three stages of development. The amounts of the following proteins were increased in stable atherosclerotic plaques at the stage of lipidosis and fibrosis: vimentin, tropomyosin β-chain, actin, keratin, tubulin β-chain, microfibril-associated glycoprotein 4, serum amyloid P-component, and annexin 5. In plaques at the stage of fibrosis and calcification, the amounts of mimecan and fibrinogen were increased. In unstable atherosclerotic plaque of the necrotic–dystrophic type, the amounts of human serum albumin, mimecan, fibrinogen, serum amyloid P-component and annexin were increased. Conclusion: This proteomic study identifies the proteins present in atherosclerotic plaques of coronary arteries by comparing their proteomes at three different stages of plaque development during coronary atherosclerosis.

## 1. Introduction

Proteomic studies have contributed substantially to the research on the pathogenesis of cardiovascular diseases, thus laying the foundation for the discovery of novel molecular targets and novel biomarkers of the risk of cardiovascular diseases and their complications [1,2]. Mass-spectrometric identification of proteins by means of peptide maps enables investigators to overcome the difficulties related to the wide variation of protein amounts in samples and to post-translational modifications [3].

Studies in the field of coronary atherosclerosis etiology and pathogenesis, which predetermine coronary atherosclerosis complications, are currently relevant because of the high prevalence and mortality rate of this disease. Several proteomic studies on human atherosclerotic plaques have been conducted to date. By mass-spectrometric analysis, Herrington et al. have investigated the human arterial proteome and proteomic features. They uncovered substantial differences in the prevalence of a mitochondrial protein, tumor necrosis factor α, insulin receptor, and PPAR-α and -γ between coronary and aortic samples and between atherosclerotic and healthy tissues [4]. The study by Han and coworkers, in patients with coronary atherosclerosis, detected the upregulation of an enzyme called CDK9, which correlated with high quantities of CD14-like antigen and monocytes/macrophages in the atherosclerotic lesion [5]. Lepedda et al. have applied proteomic analysis to atherosclerotic plaque homogenates that were obtained during endarterectomy in patients with atherosclerosis of the carotid arteries. These authors detected 33 proteins differentially expressed in stable and unstable plaques. In unstable plaques, they uncovered consistent upregulation of the proteins ferritin, SOD2, and fibrinogen (fragment D) and downregulation of glutathione S-transferase and SOD3 [6]. In a comparison of the proteomic profiles of homogenates of stable and unstable atherosclerotic plaques from the same person, some researchers have discovered that ferritin and fibrinogen are upregulated in unstable plaques, whereas in stable atherosclerotic plaques, there are high levels of apo-E, actin, and L-lactate dehydrogenase B [7].

To study the involvement of proteins in the pathological process of coronary atherosclerosis, it is important to investigate the specific relations between proteins characteristic of different developmental stages of atherosclerotic lesions in coronary arteries. In addition to known lipid and inflammatory molecules, some proteins can affect the development of atherosclerotic lesions into unstable plaques. A time-course study of the proteomic profile of the vascular wall during coronary atherosclerosis may help to discover possible diagnostically significant protein patterns or potential biomarkers of the disease and to develop new approaches for the diagnosis of coronary atherosclerosis and its complications.

The aim of this study was to investigate via proteomic technologies changes in the proteomic profile of atherosclerotic plaques at different stages of development in coronary arteries during coronary atherosclerosis.

## 2. Methods

The study protocol was approved by the local Ethics Committee of the Institute of Internal and Preventive Medicine (a branch of the Institute of Cytology and Genetics, the Siberian Branch of the Russian Academy of Sciences, Novosibirsk, Russia, protocol №4, approval date: 26 September 2017). Written informed consent to be examined and to participate in the study was obtained from each patient. All the authors meet the International Committee of Medical Journal Editors (ICMJE) criteria for authorship for this manuscript, take responsibility for the integrity of the work as a whole, and have given final approval to the version to be published.

### 2.1. Sample Collection

The object of research consisted of the samples of atherosclerotic plaque tissues containing the intima–media layer of coronary arteries. All the samples were collected from patients with well-pronounced coronary atherosclerosis, who underwent coronary bypass surgery, during which it was deemed necessary to perform endarterectomy from coronary arteries. All the participants signed informed-consent forms regarding their enrollment in the study.

To minimize differences in clinical characteristics among the groups, the samples were collected from 15 patients with similar clinical characteristics: men, age 62.89 ± 6.97 (49/71) years (M ± SD (min/max)), body-mass index 28.5 ± 4.1 (21.11/33.74), diagnosis: angina pectoris III FC; two people in each group had myocardial infarction in the past, none of the patients had a history diabetes type 2, and all were non-smokers. Exclusion criteria were as follows: myocardial infarction less than 6 months before the study, acute chronic infectious and inflammatory diseases or their deterioration, renal failure, active liver disease, cancer, and hyperparathyroidism. The same amount of material (five plaques from five patients) was collected in each group.

After morphological and histological analyses, all samples were categorized as stable or unstable atherosclerotic plaques, according to the criteria of Waksman and Seruys (2004) and Shah (2005) [8,9]. For the proteomic experiment, we prepared pools of homogenates of atherosclerotic plaques at different stages of development: 1) a pool of five homogenates of stable atherosclerotic plaques at the stage of lipidosis and fibrosis, 2) a pool of five homogenates of stable atherosclerotic plaques at the stage of fibrosis and calcification, 3) a pool of five homogenates of unstable atherosclerotic plaques of the necrotic–dystrophic type.

### 2.2. Sample Preparation and Two-Dimensional Electrophoresis

Pieces of tissue were ground in liquid nitrogen and lysed in electrophoresis buffer, incubated for 20 min at room temperature, then disrupted with an ultrasonicator (Ultrasonic CPX 130; Cole Parmer, Vernon Hills, IL, USA) for 30 s on ice. The suspension was centrifuged for 30 min at 10 °С and 16,100× *g*, and the protein concentration was measured by the QuickStart™ Bradford protein assay (Bio-Rad, Hercules, CA, USA).

The proteins from atherosclerotic plaques were separated by two-dimensional electrophoresis. For this procedure, sample pools were prepared that corresponded to three stages of atherosclerotic plaque development. To this end, equal amounts of proteins (100 μg) from five samples at each stage were mixed. Next, 100 μg of proteins of each pool was loaded on strips 17 cm-long, pH range 4–7 (ReadyStrip™ IPG Strip, Bio-Rad, Hercules, CA, USA). Five technical replicates were analyzed for each pool. The strips were rehydrated in electrophoresis buffer, 300 μL of each sample was loaded on the strips, and separation lasted for 12 at 50 mA per strip at 20 °С. Before separation in the second direction, the strips were stored at −80 °С and then equilibrated in a buffer supplemented with 130 mM dithiothreitol for 15 min and then in the above buffer supplemented with 200 mM iodoacetamide for additional 15 min. The strips were placed on a 12% polyacrylamide gel. The transfer of proteins into the gel was performed at 16 mA current, with subsequent separation of proteins at 25 mA per gel.

The gels were stained with a fluorescent dye, SyproRuby (Bio-Rad, Hercules, CA, USA) in a DodecaGelStainer camera (Bio-Rad) according to the manufacturer’s instructions. The stained gels were visualized by means of the VersaDoc MP4000 imaging system (Bio-Rad). Gel images were analyzed by the PDQuest software, version 8.1.0 (Bio-Rad). The gel with the greatest amount and the best resolution of protein spots was chosen as a master gel, relative to which all the protein spots were compared and matched among gels. To identify the differences between the stages, a comparison of the amount of proteins in each spot, expressed in relative units of intensity, was carried out. Correct matching of specific protein spots between gels was verified visually, with corrections if necessary.

The spots were cut out of the gels using an EXQuestSpotCutter (Bio-Rad), and the gel pieces were washed with distilled water and incubated in a 50 mM solution of ammonium bicarbonate in 50% acetonitrile for 20 min at room temperature. After that, the gels were dried in acetonitrile, and the proteins were digested in-gel with trypsin via rehydration of the gel with a solution of modified porcine trypsin (TrypsinGold, MassSpectrometryGrade, Promega, Madison WI, USA) at 15 μg/mL in50 mM ammonium bicarbonate. The hydrolysis lasted for 12 h at 37 °С.

### 2.3. Mass Spectrometry Analysis

Isolation and purification of tryptic peptides was carried out using Millipore ZIP TIP C18 (Millipore, Burlington MA, USA). Purified peptides were mixed with a saturated matrix solution, α-cyano-4-hydroxy-cinnamic acid (Bruker, Bremen, Germany) in 70% acetonitrile (Merck, Darmstadt, Germany) with 0.1% trifluoroacetic acid (Sigma, Sigma-Aldrich St. Louis, MO, USA). An aliquot (1 µl) of the resulting mixture was applied to a metal target of the mass spectrometer and air-dried. The measurements were carried out on an Ultraflex III instrument (Bruker, Bremen, Germany) using the following parameters: accelerating voltage: 25 kV, voltage reflectron: 26.3 kV; mass range 500–3500 Da, and delayed extraction 200 ns. Instrument calibration was performed using a standard type of hydrolysate of bovine serum albumin (Bruker, Bremen, Germany) at the following masses (Da): 937.48, 1163.63, 1283.71, 1305.71, 1399.69, 1439.81, 1479.79, 1567.74, 1639.93, 1724.84, 1880.92, 1907.92, 2045.02.

Proteins were identified via their tryptic mass map by the search algorithm Mascot (available online: http://matrixscience.com/home.html (6 October 2019). The search parameters were as follows: mass error ±0.05 Da, missing cleavages: one, modifications: oxidation of methionine and carbamidomethylation of cysteines, and database: SwissProt.

To adjust the data for differences in the intensity and size of the protein spots as a result of pipetting variation, the local regression model was chosen as a normalization method. Five technical replicates of each pool (corresponding to each stage of atherosclerotic plaque development) were analyzed, and statistical significance was assumed at *р* < 0.05.

## 3. Results

During the comparison of the gels, the most abundant protein spots were selected for identification by MALDI mass spectrometry. The proteomic analysis uncovered groups of proteins (*n* = 55) that were differentially expressed by more than twofold (*p* < 0.05) between pools of homogenates from the three stages of atherosclerotic plaque development. Thirty spots were identified; the results of mass-spectrometric identification are given in Table 1.

Some groups of spots contained isoforms of the same protein, with different weight and pI, for example serum albumin, vimentin, fibrinogen β chain, microfibril-associated glycoprotein 4 (MAGP-4), tubulin β chain. A mix of different isoforms of actin (alpha cardiac muscle 1, aortic smooth muscle, cytoplasmic 1, cytoplasmic 2) was identified in some spots. A mix of tubulin β chain isoforms was found in spot 7. Table 1 shows only the isoforms with the highest values of sequence coverage and score.

Increased amounts were noted for the cytoskeletal proteins vimentin, tropomyosin β chain, aortic smooth muscle actin, α cardiac muscle actin 1, tubulin β chain, MAGP-4, and keratin in the sample of stable atherosclerotic plaques at the stage of lipidosis and fibrosis. In addition, the maximum amounts of serum amyloid P-component (SAP) and annexin A were identified at the stage of lipidosis and fibrosis. The amounts of SAP and annexin 5 were significantly reduced in the sample at the stage of fibrosis and calcification, but at the stage of unstable plaques, the amounts of these proteins increased again.

The relative amount of proteins in each spot was determined by the PDQuest program in relative units of intensity. Table 2 shows the averaged amounts of protein at the different stages of atherosclerotic plaques development; the protein isoforms are grouped together and differ only in pI.

Increased amounts were identified for fibrinogen β chain and mimecan in the sample of stable atherosclerotic plaques at the stage of fibrosis and calcification. Increased amounts were noted for serum albumin, mimecan, fibrinogen, SAP, and annexin 5 in plaques of the necrotic–dystrophic type at the stage of unstable atherosclerotic plaques.

Thus, stable plaques at the stage of lipidosis and fibrosis are characterized by increased amounts of vimentin, tropomyosin β chain, actin, tubulin β chain, MAGP-4, keratin, SAP, and annexin A. Stable plaques at the stage of fibrosis and calcification are characterized by increased amounts of fibrinogen β chain and mimecan and decreased amounts of SAP and annexin A. Unstable atherosclerotic plaques are characterized by increased amounts of serum albumin, mimecan, fibrinogen, SAP, and annexin 5.

## 4. Discussion

Actin is a contractile catalytically inactive protein, existing in the forms of globular (monomeric) G-actin (43 kDa) and double-helical filaments called F-actin. Actin is functionally associated with tropomyosin and troponins. Tropomyosin (TM) is a regulatory fibrillar protein (70 kDa) consisting of two intertwined α-helices, which forms one complex with F-actin and ensures its stability. The stoichiometry of the TM–F-actin complexes corresponds to a molar ratio of 1. TM participates in the calcium-dependent regulation of muscle contraction [10]. Mutations in the tropomyosin 1 gene can cause hereditary cardiomyopathies, hypertrophy of the left ventricle, or disturbances of the diastolic function in the absence of hypertension and aorta stenosis [11].

The cytoskeleton proteins include tubulin, an important component of microtubules. Microtubules are involved in the implementation of the functions of endothelial cells, such as maintenance of cell shape, mitosis, intracellular transport, adhesion, and migration. Posttranslational modifications of tubulin, such as deacetylation, phosphorylation, and polyglutamation, are involved in the regulation of microtubule dynamics [12]. Oxidative stress and chemical modification of tubulin lead to the reorganization of endothelial microtubules. These changes destabilize vascular integrity and increase permeability, which finally results in increased cardiovascular risk [13].

Our study demonstrated a significant increase in tubulin and MAGP-4 content in the sample of atherosclerotic plaques at the stage of lipidosis and fibrosis compared with the sample of stable plaques at the stage of fibrosis and calcification and with the sample of unstable plaques.

The family of microfibril-associated glycoproteins (MAGP) consists of proteins of the extracellular matrix that affect the functionality of the cell. Gene mutations of these proteins are associated with aortic aneurysm, and increased expression of MAGP-2 has been proposed as an independent prognostic biomarker in ovarian cancer [14]. MAGP-4 has been proposed as one of the direct serum markers for assessing the development of liver fibrosis [15]. The expression levels of MAGP-4 and vimentin were found to be reduced in hemorrhagic atherosclerotic plaques of the carotid arteries, compared to fibrous plaques [16].

Vimentin (55 kDa) is an intermediate filament protein and a specific marker of changes in the molecular phenotype of epithelial cells; in fact, it is expressed de novo during phenotype change toward a mesenchymal phenotype. The dynamic nature of vimentin is important for alterations of cell shape, and this protein ensures structural integrity of the cell and its resistance to mechanical stress. It has been shown that cells devoid of vimentin are extremely sensitive to mechanical damage [17,18]. After damage to epithelial cells, vimentin plays the main role in the recovery of cell function [19]. Human vimentin contains a single cysteine residue at position 328, which can serve as a sensor of oxidative stress. Mutation of this residue increases the resistance of vimentin to oxidative agents and facilitates its remodeling. In addition, zinc, which is functionally linked to vimentin, increases its resistance to oxidative stress [20]. In a study in mice, it was found that vimentin suppresses the formation of reactive oxygen species, whereas vimentin deficiency in macrophages disrupts endocytosis and the inflammatory response, i.e., vimentin participates in the regulation of macrophage-mediated inflammation during atherogenesis [21].

In our study, it was demonstrated that stable atherosclerotic plaques at the stage of lipidosis and fibrosis were characterized by increased amounts of cytoskeletal proteins such as actin, tropomyosin, tubulin, MAGP-4, and vimentin; this alteration is apparently associated with cellular migration to the subendothelial space after damage to the vessel wall (during subsequent inflammation). Meanwhile, stable atherosclerotic plaques at the stage of lipidosis and fibrosis contained greater amounts of these proteins. Evidently, this phenomenon may be explained by the fact that a stable atherosclerotic plaque at the stage of lipidosis and fibrosis is more common at the earlier stages of the atherosclerotic process.

Type I keratins are cytokeratins (40–70 kDa) that constitute intermediate filaments of the cytoskeleton and are present in all epithelial cells of mammals. The expression of cytokeratins located in smooth muscle cells is significantly higher in carotid arteries with atherosclerosis in comparison with healthy carotid arteries [22]. Cytokeratin I in endothelial cells serves as a receptor for many active molecules such as fatty acid-binding protein, whereas oxidative stress up regulates cytokeratin I [23].

Our study indicates that the amount of keratin was significantly increased in the samples of stable atherosclerotic plaques at the stage of lipidosis and fibrosis, as compared with unstable atherosclerotic plaques. This finding does not contradict the above-mentioned properties of cytokeratin in relation to aberrations in endothelium function and oxidative changes that are typical for earlier stages of atherosclerotic lesion development, that in our study, we suggested for stable atherosclerotic plaques.

Our results are consistent with those of Tu et al., who have reported substantial upregulation of the proteins tropomyosin, actin, and keratinin in the carotid artery and middle cerebral artery in rabbits with hyperlipidemia and with insignificant atherosclerotic changes [24].

Fibrinogen is a known acute-phase protein, a glycoprotein involved in the final stage of coagulation. Fibrinogen was present in the samples of atherosclerotic plaques at all stages of development, but the amount of this protein increased significantly (more than two fold) in stable atherosclerotic plaques at the stage of fibrosis and calcification and in a sample of unstable plaques compared to the sample of plaques at the stage of lipidosis and fibrosis.

In addition, we found the maximum amount of SAP and annexin A5 in the sample of atherosclerotic plaques at the stage of lipidosis and fibrosis. In this case, the contents of these proteins were significantly reduced at the stage of fibrosis and calcification but increased again at the stage of unstable plaques.

SAP is an acute-phase protein, a member of the pentraxin family of serum proteins. SAP plays a significant role in the biological processes of the cardiovascular system, such as inflammation and fibrosis. The amount of SAP in tissues increases in degenerative aortic stenosis; in addition, SAP levels in plasma are positively associated with cardiovascular diseases in the elderly [25]. In a study on apolipoprotein E-deficient (Apoe−/−) mice, intraperitoneal injection of SAP was shown to inhibit atherosclerosis in these animals [26], expression of SAP increased in hemorrhagic atherosclerotic plaques of carotid arteries, compared with fibrous plaques [16].

Annexin 5 belongs to a group of annexin proteins with structural similarity and functional ability to bind phospholipids in the presence of calcium ions. Annexin 5 is found in the vascular endothelium and has anti-inflammatory, anticoagulant, and anti-apoptotic effects by binding phosphatidylserine molecules [27]. In the study of Lee at al., it was shown that the amount of annexin 5 in plasma increased significantly after plaque disruption [28].

At present, our data do not allow us to answer the question of why the amount of these proteins decreased in stable atherosclerotic plaques at the stage of fibrosis and calcification but was restored in unstable plaques. In addition, the sample of atherosclerotic plaques at the fibrosis and calcification stage was characterized by a high content of mimecan relative to the plaques at the lipidosis and fibrosis stage and relative to the sample of unstable plaques.

Mimecan (or osteoglycin) is a major component of the extracellular matrix. Mimecan is involved in the pathogenesis of atherosclerosis by regulating proliferation, apoptosis, and migration of vascular smooth muscle cells [29]. Proteomic analysis showed that the expression of this protein was reduced in atherosclerotic plaques. In addition, mimecan can play a key role in tumorigenesis [30]. In a study by Seki et al., it was shown that the amount of mimecan (osteoglycin) in the serum of patients with coronary atherosclerosis increased, but high levels of osteoglycin were not correlated with the severity of the disease. Since in patients with complex lesions its level was lowered, it was suggested that osteoglycin plays a role in coronary plaque stabilization [31]. The high content of mimecan in the sample of stable atherosclerotic plaques (fibrosis and calcification) in our study confirms these findings. In a study by Malaud et al., it was suggested that a decrease in osteoglycin expression in hemorrhagic plaques can lead to instability of the plaque [16]. In a study examining the prognostic value of certain biomarker proteins for coronary artery disease patients, circulating osteoglycin, whose expression was elevated in vulnerable atherosclerotic plaques, was suggested as a promising biomarker of adverse cardiovascular events [32].

Human serum albumin (HSA) is the main protein of human plasma. HSA is synthesized in the liver, serves as an amino acid reserve for protein synthesis, and performs a transport function in the blood. HSA has antioxidant activity because of its ability to bind reactive oxygen species [33]. HSA and Fetuin A inhibit the process of vascular calcification by binding to calcium phosphate particles, thereby slowing their intracellular dissolution [34]. Previous studies showed that the prooxidant environment of atherosclerotic plaques could modify plaque-filtered HSA by protein-SH group oxidation, contributing to plaque progression. At the same time, the plaques contain three times more oxidized HSA than serum [35].

Dirajlal-Fargo and coworkers have demonstrated that a low concentration of HSA in the blood is a predictor of atherosclerosis in blood vessels regardless of the traditional risk factors and statin therapy in patients with HIV infection. Furthermore, it has been shown that HSA is associated with markers of systemic inflammation and hypercoagulation (interleukin 6, tumor necrosis factor α, С-reactive protein, fibrinogen, and D-dimer). The pathophysiological mechanism underlying this association—according to thwse authors—is the ability of HSA to bind many ligands, including proatherogenic ones, thereby preventing their contribution to oxidative stress [36]. In the “Framingham Offspring” study, which included 4506 people and continued for 22 years, it was proven that HSA is an independent predictor of the first myocardial infarction [22].

The mechanism triggering the development of myocardial infarction involves a tear or crack in the thin cover of an (predominantly) unstable atherosclerotic plaque; therefore, the results of our study do not contradict the above-mentioned data. We detected increased amount of HSA in the sample of unstable atherosclerotic plaques of the necrotic–dystrophic type. An unstable atherosclerotic plaque is characterized by overexpression of various proatherogenic factors and ligands, thus possibly drawing HSA from the blood plasma to the atherosclerotic lesions.

## 5. Conclusions

In a proteomic experiment, we demonstrated that early phases of coronary atherosclerosis at the stage of stable atherosclerotic plaques (lipidosis and fibrosis) show increased amounts of cytoskeletal proteins (actin, tropomyosin β chain, vimentin, keratin, tubulin β chain, and MAGP-4), SAP, and annexin 5.

The progression of the atherosclerotic process from stable atherosclerotic plaques to the stage of fibrosis and calcification leads to a decrease in the concentration of these proteins and to a significant increase in the levels of mimecan and fibrinogen.

At the stage of an unstable atherosclerotic plaques of the necrotic–dystrophic type, an increased amount of HSA is observed. It is possible that the high concentration of HSA in an atherosclerotic plaque correlates with the instability of the atherosclerotic plaque. In addition, unstable plaques are characterized by the preservation of a high content of mimecan and fibrinogen and the re-establishment of the previous high level of SAP and annexin 5.

This proteomic study identifies the proteins of atherosclerotic plaques in coronary arteries by comparing plaque proteomes at three different stages of plaque development during coronary atherosclerosis. These results require validation in further studies with large groups to confirm the prognostic value of these biomarkers and their association with instable human coronary artery plaques.

## Figures and Tables

**Table 1:** Mass-spectrometric identification of proteins in the pools of homogenates of atherosclerotic plaques.

№	ID (NCBI)	Protein Name	pI/Mass (Da)	Sc %	Score
1	ALBU_HUMAN	Serum albumin	5.92/71317	21	97
2	ALBU_HUMAN	Serum albumin	5.92/71317	23	112
3	ALBU_HUMAN	Serum albumin	5.92/71317	33	185
4	VIME_HUMAN	Vimentin	5.06/53676	50	252
5	VIME_HUMAN	Vimentin	5.06/53676	60	299
6	VIME_HUMAN	Vimentin	5.06/53676	56	189
7	TBB4B_HUMAN	Tubulin β -4B chain	4.79/50255	21	70
	TBB2A_HUMAN	Tubulin β -2A chain	4.78/50274	19	58
	TBB2B_HUMAN	Tubulin β -2B chain	4.78/50377	19	58
	TBB5_HUMAN	Tubulin β chain	4.78/50095	17	57
8	ACTC_HUMAN	Actin, α cardiac muscle 1	5.23/42334	50	125
	ACTA_HUMAN	Actin, aortic smooth muscle	5.23/42381	50	125
9	ACTC_HUMAN	Actin, α cardiac muscle 1	5.23/42334	44	127
	ACTA_HUMAN	Actin, aortic smooth muscle	5.23/42381	26	85
10	ACTC_HUMAN	Actin, α cardiac muscle 1	5.23/42334	55	128
	ACTA_HUMAN	Actin, aortic smooth muscle	5.23/42381	55	128
11	ACTB_HUMAN	Actin, cytoplasmic 1	5.29/42052	61	157
	ACTG_HUMAN	Actin, cytoplasmic 2	5.29/42108	61	157
12	FIBB_HUMAN	Fibrinogen β chain	8.54/56577	21	94
13	FIBB_HUMAN	Fibrinogen β chain	8.54/56577	35	158
14	FIBB_HUMAN	Fibrinogen β chain	8.54/56577	42	191
15	TPM2_HUMAN	Tropomyosin β chain	4.66/32945	38	117
16	MFAP4_HUMAN	Microfibril-associated glycoprotein 4	5.38/28972	24	80
17	MFAP4_HUMAN	Microfibril-associated glycoprotein 4	5.38/28972	24	80
18	MFAP4_HUMAN	Microfibril-associated glycoprotein 4	5.38/28972	22	57
19	MIME_HUMAN	Mimecan	5.46/34243	46	180
20	ANXA5_HUMAN	Annexin A5	4.94/35971	58	175
21	K1C9_HUMAN	Keratin, type I cytoskeletal 9	5.14/62255	32	79
22	SAMP_HUMAN	Serum amyloid P-component	6.1/25485	28	80
23	SAMP_HUMAN	Serum amyloid P-component	6.1/25485	33	77

Note: sc: sequence coverage; score: Mascot statistical score.

**Table 2:** Comparison of the amount of proteins in the pools of homogenates of atherosclerotic plaques (maximum in bold).

Number	Id (NCBI)	Protein Name	Amount of Protein, Relative Units of Intensity, *10^5^		
			StL	StF	Ns
1–3	ALBU_HUMAN	Serum albumin	4.3	12.3	**46.3**
4–6	VIME_HUMAN	Vimentin	**10.1**	2.4	4.1
7	TBB5_HUMAN	Tubulin β chain	**2.5**	1.4	1.1
8–10	ACTC_HUMAN	Actin, α cardiac muscle Actin, aortic smooth muscle	**84**	29.2	33.4
11	ACTB_HUMAN	Actin, cytoplasmic Actin, cytoplasmic 2	**91.3**	18.4	37.7
12–14	FIBB_HUMAN	Fibrinogen β chain	1.3	**3.2**	2.9
15	TPM2_HUMAN	Tropomyosin βchain	**40.3**	2.0	2.0
16–18	MFAP4_HUMAN	Microfibril-associated glycoprotein 4	**22.4**	4.5	3.2
19	MIME_HUMAN	Mimecan	26.5	**126.5**	55.4
20	ANXA5_HUMAN	Annexin A5	**2.8**	0.7	2.2
21	K2C1_HUMAN	Keratin, type I cytoskeletal 9	**6.4**	-	1.7
22–23	SAMP_HUMAN	Serum amyloid P-component	**25.3**	5.9	22.2

Note: StL: stable atherosclerotic plaques at the stage of lipidosis and fibrosis (1); StF: stable atherosclerotic plaques at the stage of fibrosis and calcification (2); Ns: unstable atherosclerotic plaques of the necrotic-dystrophic type (3). *: The unit of measure provided by the PDQuest program.
